# Low Incidence of Fatigue after Hypofractionated Stereotactic Body Radiation Therapy for Localized Prostate Cancer

**DOI:** 10.3389/fonc.2012.00142

**Published:** 2012-10-17

**Authors:** Chiranjeev Dash, Kristina Demas, Sunghae Uhm, Heather N. Hanscom, Joy S. Kim, Simeng Suy, Kimberly M. Davis, Jennifer Sween, Sean Collins, Lucile L. Adams-Campbell

**Affiliations:** ^1^Department of Oncology, Lombardi Comprehensive Cancer Center, Georgetown UniversityWashington, DC, USA; ^2^Department of Radiation Medicine, Georgetown University HospitalWashington, DC, USA

**Keywords:** fatigue, prostate cancer, stereotactic body radiation therapy, cyberknife, radiosurgery, total monitor unit

## Abstract

**Background:** Fatigue is a common side effect of conventional prostate cancer radiation therapy. The increased delivery precision necessitated by the high dose per fraction of stereotactic body radiation therapy (SBRT) offers the potential of reduce target volumes and hence the exposure of normal tissues to high radiation doses. Herein, we examine the level of fatigue associated with SBRT treatment. **Methods:** Forty patients with localized prostate cancer treated with hypofractionated SBRT, and a minimum of 12 months follow-up were included in this analysis. Self-reported fatigue and other quality of life measures were assessed at baseline and at 1, 3, 6, 9, and 12 months post-SBRT. **Results:** Mean levels of fatigue were elevated at 1 month post-SBRT compared to baseline values (*P* = 0.02). Fatigue at the 3-month follow-up and later were higher but not statistically significantly different compared to baseline. African-American patients reported higher fatigue post-SBRT than Caucasian patients. Fatigue was correlated with hormonal symptoms as measured by the Expanded Prostate Cancer Index Composite (EPIC) quality of life questionnaire, but not with urinary, bowel, or sexual symptoms. Age, co-morbidities, smoking, prostate specific antigen (PSA) levels, testosterone levels, tumor stage, and treatment variables were not associated with fatigue. **Conclusion:** This is the first study to investigate fatigue as a side effect of SBRT. In contrast to standard radiation therapy, results suggest SBRT-related fatigue is short-term rather than a long-term side effect of SBRT. These results also suggest post-SBRT fatigue to be a more frequent complication in African-Americans than Caucasians.

## Introduction

Fatigue limits a patient’s ability to care for themselves and decreases their quality of life (Hickok et al., 2005; Ryan et al., 2007). Unfortunately, fatigue is a common radiation therapy side effect (Jereczek-Fossa et al., 2002) even in patients with localized prostate cancer (Walker et al., 1996). Currently, the etiology of radiation therapy-related fatigue is poorly understood and likely multi-factorial (Ryan et al., 2007). Typically, fatigue begins shortly after the initiation of treatment and can take months to resolve following the completion of treatment (Hickok et al., 2005; Kyrdalen et al., 2010). Chronic fatigue can occur and may be related to patient specific factors or treatment-related side effects (Ryan et al., 2007). Radiation-induced fatigue is treatment volume-dependent with larger fields causing increased fatigue (Beard et al., 1997). The level of fatigue is also dependent on the treatment site, with pelvic irradiation inducing testosterone reductions that may be partially responsible for radiation-induced fatigue (Pickles and Graham, 2002; Oermann et al., 2011).

Standard radiation therapy for clinically localized prostate cancer entails 8–9 weeks of daily low dose (1.8–2.0 Gy fractions) radiation. This demanding schedule possibly induces fatigue independent of treatment. Recently, large radiation fraction sizes have been shown to be radiobiologically favorable compared to smaller fraction sizes in prostate cancer radiotherapy (Fowler, 2005). Typically, hypofractionated stereotactic body radiation therapy (SBRT) delivers 35–40 Gy to the prostate in four to five fractions. CyberKnife (Accuray, Inc., Sunnyvale, CA, USA) seems ideal for delivering hypofractionated SBRT with its sub-millimeter accuracy (Kilby et al., 2010). Unlike standard radiation therapy delivery systems, the CyberKnife incorporates a real-time motion tracking system that provides updated target position and corrects the targeting of the therapeutic beam during treatment. Intrafraction motion tracking allows for a reduction in the planning target volume (PTV) and potentially the dose to surrounding critical organs. These abilities allow for dose escalation within the prostate while maintaining normal tissue tolerance and thereby reducing potential side effects of radiation therapy.

Published outcomes for CyberKnife delivered SBRT suggest high biochemical control rates with acceptable toxicity a few years following treatment (King et al., 2003, 2009; Fuller et al., 2008; Friedland et al., 2009; Katz et al., 2010; Oermann et al., 2011; Jabbari et al., 2012). Indeed, recent updates have confirmed a 5-year biochemical disease-free survival in excess of 90% (Freeman and King, 2011; King et al., 2012). In addition, early quality of life data indicate that SBRT is well tolerated with declines in patient reported urinary, bowel, and sexual function similar to other radiation therapy treatments (Sanda et al., 2008). We hypothesize that reducing the length of treatment and treatment volumes, could reduce treatment-related fatigue. In this study of prostate cancer patients undergoing hypofractionated SBRT, we evaluated cancer-related fatigue at baseline (prior to treatment) and at 1, 3, 6, 9, and 12 months post-treatment.

## Materials and Methods

### Study population

Patients included in this retrospective Medstar-Georgetown University institutional review board approved study were treated and followed-up from January 2010 to November 2011 with hypofractionated SBRT for clinically localized prostate adenocarcinoma. The patient eligibility criteria were: (1) histologically confirmed prostate cancer; (2) age 75 or less; (3) a minimum of 12 months follow-up; and (4) completion of the Functional Assessment of Cancer Therapy (FACIT)-Fatigue survey (Cella et al., 1993; Lai et al., 2003; Davis et al., 2008), Expanded Prostate Cancer Index Composite (EPIC-26; Wei et al., 2000), and the American Urological Association (AUA) prostate symptom questionnaires. Informed consent was obtained from all participants prior to participation in the study.

### Treatment

All patients received CyberKnife delivered SBRT as previously described (Oermann et al., 2011). Briefly, a linear accelerator mounted on a flexible robotic arm delivered a few hundred unique treatment beams in a non-isocentric manner via circular collimators. The gross tumor volume (GTV) included the prostate and the proximal seminal vesicles. The PTV equaled the GTV expanded 3 mm posteriorly and 5 mm in all other dimensions. The prescribed dose was 35 or 36.25 Gy in five fractions of 7–7.25 Gy over 1–2 weeks. The prescription isodose line was limited to >75%.

### Assessments

Patients were evaluated at 1, 3, 6, 9, and 12 months post-SBRT. Prostate specific antigen (PSA) and testosterone levels were recorded, toxicity assessed, and fatigue and health related quality of life (HRQOL) questionnaires completed at baseline and each follow-up. Fatigue was measured using the FACIT-Fatigue survey managed and distributed by FACIT.org^1^ The FACIT-Fatigue is a 13-item subscale developed to identify a finite set of concerns specific to fatigue. Fatigue scores for the 13-item fatigue scale range from 0 to 52, where lower scores indicate low energy or higher fatigue. Similarly, higher scores indicate higher energy and lower fatigue.

Health related quality of life (HRQOL) was measured using two instruments – the EPIC-26 survey and the AUA prostate symptom survey. The EPIC-26 is a 26-item short form version of the original EPIC survey designed to evaluate patient function and bother after prostate cancer treatment (Wei et al., 2000). The survey asks questions and provides a score associated with the following five domains of HRQOL specific to prostate cancer therapy: urinary irritative/obstructive, urinary incontinence, bowel, sexual, and hormonal domains.

The AUA prostate symptom index is prostate function survey developed and validated by the Multidisciplinary Measurements Committee of the AUA. The 7-item survey assesses non-specific urinary symptoms (incomplete emptying, frequency, intermittency, urgency, weak stream, straining, and nocturia) associated with clinical benign prostatic hypertrophy. A high score indicates greater symptom bother and lower HRQOL.

In addition to information on fatigue and HRQOL, data on the following covariates were also collected: age, race, co-morbidities including history of HIV and other cancers, smoking status, PSA levels, testosterone levels, AJCC/UICC TNM stage, and Gleason score.

#### Statistical analysis

Baseline characteristics of the study participants were summarized using frequencies and means. Fatigue scores (FACIT-Fatigue scale) were calculated using guidelines provided by the FACIT Measurement System (FACIT.org). Domain scores for the EPIC-26 survey were calculated using the information provided in the University of Michigan’s EPIC website^2^. AUA symptom score was calculated by summing the responses for the 7 items in the score for each participant.

Correlations between the FACIT-Fatigue scores and the HRQOL measures were tested using non-parametric (Spearman correlation coefficient) methods. Associations of fatigue scores prior to SBRT (at baseline) with selected variables (age, race, smoking status, HIV status, other cancer diagnosis, tumor stage, PSA at baseline, and testosterone levels at baseline) were investigated using multiple linear regression models. Linear generalized estimating equations (GEE) models were used to determine whether post-SBRT fatigue scores (at 1, 3, 6, 9, and 12 months post- treatment) were significantly different from baseline scores. Repeat fatigue score measurements for an individual over time were accounted for using an autoregressive model of correlation that assumes a higher degree of correlation between measurements conducted closer in time than those that are farther apart. The age-adjusted fatigue scores were derived using the least square means in the GEE models that included age as a fixed effect. *P*-values for the difference between pre- and post-SBRT fatigue scores were adjusted for the following covariates: age, race, co-morbidities including history of HIV and other cancers, smoking status, PSA levels, testosterone levels, and tumor stage. Clinical significance of the change in scores from baseline at each follow-up visit was defined as change exceeding half the standard deviation of the baseline score. The GEE models were further stratified by race (African-American and Caucasian) to determine whether there was any evidence of effect modification by these variables on the fatigue scores pre- and post-SBRT. Statistical significance of the effect modification by race was determined by including a multiplicative (race × treatment) variable in the GEE model. All statistical analyses were conducted using SAS v. 9.3 (SAS Systems, Inc.).

## Results

Forty patients with a minimum of 12 months follow-up met the eligibility criteria and are the subject of this analysis. The patient characteristics are presented in Table 1. The majority of the patients were either African-American (50%) or Caucasian (45%). Although all patients reported some type of co-morbidity, only 2 (4%) were HIV positive and 4 (10%) reported a history of second cancer in addition to the prostate cancer. The majority of the patients were non-smokers (75%). As expected, given the tumor stage eligibility for SBRT therapy, all patients were either clinically T1 (65%) or T2(35%) at the time of diagnosis. The majority of patients (94%) had Gleason scores of 6 (45%) or 7 (49%); 1 (3%) patient had Gleason scores of 8. Mean baseline PSA and testosterone at diagnosis were 10.05 ng/ml and 279.26 ng/dL, respectively. Treatment variables are presented in Table 2.

**Table 1:** **Patient characteristics**.

Characteristic	*N* (%)
Age, in years; mean (SD)	70.13 (6.60)
Race
Caucasian	18 (45)
African-American	20 (50)
Other	2 (5)
Smoker
No	30 (75)
Yes	10 (25)
HIV positive
No	38 (95)
Yes	2 (5)
Other cancer
No	36 (90)
Yes	4 (10)
Tumor stage*
T1c	26 (65)
T2a	8 (20)
T2b	4 (10)
T2c	2 (5)
Gleason score
2 + 3	1 (3)
3 + 3	18 (45)
3 + 4	14 (35)
4 + 3	6 (14)
4 + 4	1 (3)
PSA at baseline, in ng/ml; mean (SD)	10.05 (22.93)
PSA at baseline (ng/ml)
<10	34 (85)
10–20	5 (13)
≥20	1 (2)
Testosterone at baseline, in ng/dL; mean (SD)	279.26 (176.32)

SD, standard deviation; PSA, prostate specific antigen. *Nodal (*N*) and metastasis (M) status could not be evaluated for 85% of the participants and are not presented.

**Table 2:** **Treatment variables**.

Characteristic	*N* (%)
Total SBRT dose (Gy), Mean (SD)
35.00	8 (20)
36.25	32 (80)
Isodose line (%), mean (SD)	77 (1)
Total non-zero beams, mean (SD)	249 (20)
PTV (cm^3^), mean (SD)	168 (43)
GTV (cm^3^), mean (SD)	88 (29)
Total monitor unit (MU), mean (SD)	57,285 (8,549)

*SBRT, stereotactic body radiation therapy; PTV, planning tumor volume; GTV, gross tumor volume*.

Overall questionnaire completion compliance was greater than 95%. No association with baseline fatigue scores was found between age, race, smoking status, HIV status, history of another cancer in addition to prostate cancer, baseline testosterone levels, or tumor stage. Baseline PSA levels and fatigue scores were statistically significantly associated (*P *= 0.05) with a higher baseline PSA level associated with a lower FACIT-Fatigue score (higher fatigue). Analysis of correlations between fatigue scores with the HRQOL measures (EPIC-26 domains and AUA symptom score) identified the EPIC hormone domain as the only HRQOL score significantly correlated with the FACIT-Fatigue score (Spearman ρ = 0.69, *P *< 0.0001). Lower FACIT-Fatigue scores were associated with higher reported HRQOL in the EPIC hormone domain.

Age-adjusted mean FACIT-Fatigue scores at baseline and during follow-up after CyberKnife SBRT are presented in Table 3. The mean fatigue was statistically significantly higher (lower FACIT-Fatigue scores) at the 1-month post-SBRT follow-up compared to baseline. However, the change in fatigue scores was not clinically significant as determined by greater than one-half of a standard deviation change from baseline FACIT-Fatigue scores. Although FACIT-Fatigue scores were lower at the 3, 6, 9, and 12 month follow-up visits compared to baseline, none of the differences were either clinically or statistically significant.

**Table 3:** **Age-adjusted pre- and post-SBRT fatigue scores**.

Facit-fatigue score	Mean (SE)*	*P* change from pre-SBRT^#^
Pre-SBRT	43.78 (1.43)	−
Post-SBRT (Month)
1	40.66 (1.43)	0.02
3	42.81 (1.43)	0.37
6	43.07 (1.45)	0.87
9	41.13 (1.45)	0.40
12	43.18 (1.43)	0.61

*SBRT, stereotactic body radiation therapy; SE, standard error*.

*Age-adjusted least square means.

*#*P*-values adjusted for age, race, smoking status, tumor stage, HIV status, other cancer diagnosis, baseline PSA values, and baseline testosterone values*.

**Figure 1:**
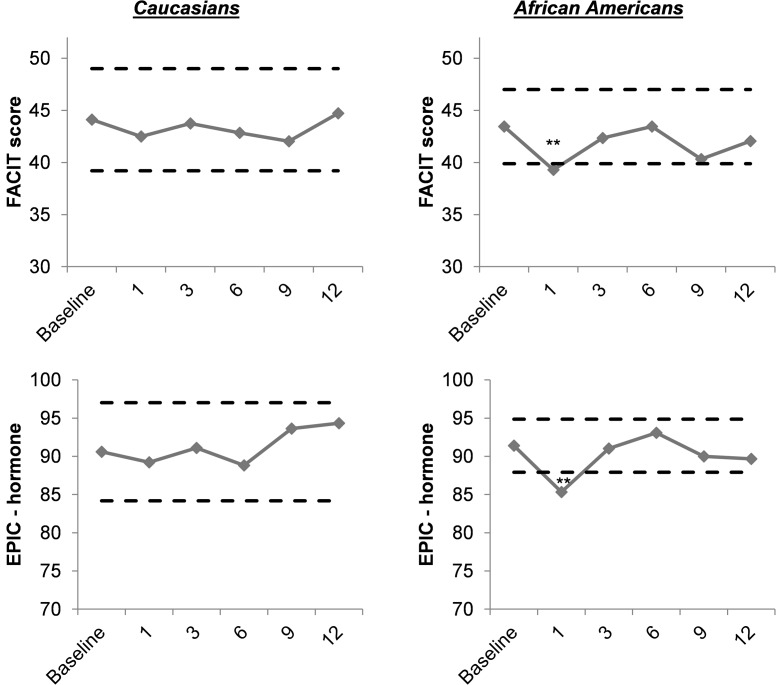
**Age-adjusted fatigue and EPIC hormone domain scores in Caucasians and African-Americans**. Dashed lines represent half standard deviation above and below the baseline scores. Change greater than half standard deviation suggests clinical significance. **Change from baseline statistically significant at <0.01 level. *Change from baseline statistically significant at <0.05 level.

The impact of race on the change in FACIT-Fatigue scores post-SBRT was compared to baseline by stratifying the age-adjusted fatigue scores by race (Figure 1). Given that the majority (95%) of patients were either African-American or Caucasian, we restricted the stratified analysis to these races. Although Caucasian patients reported lower fatigue at baseline [44.1 (9.8)] compared to African-Americans [43.5 (7.1)] the differences were not statistically significant (*P* = 0.22).

At the 1-month post-SBRT, a statistically and clinically significant increase in fatigue was observed for African-American patients but not for Caucasian patients. In addition, the 12-month post-SBRT FACIT-Fatigue score for Caucasian patients was slightly higher than the pre-treatment score but for African-Americans post-SBRT fatigue scores consistently remained low compared to the baseline.

The results of the EPIC hormone domain analysis were similar to the race-stratified results for FACIT-Fatigue scores (Figure 1). African-American patients were more likely to report a lower HRQOL on the hormone domain following SBRT than Caucasian patients. The difference in the hormone score was statistically and clinically significant between baseline and 1-month follow-up for African-American patients but not for Caucasian patients. In the multivariate models, only SBRT was significantly associated with fatigue. Age, co-morbidities including history of HIV and other cancers, smoking status, PSA levels, testosterone levels, tumor stage, and treatment variables were not associated with FACIT-Fatigue scores after adjusting for SBRT.

## Discussion

Fatigue was not a major side effect for prostate cancer patients undergoing hypofractionated SBRT. Although fatigue at 1-month post-SBRT was higher than baseline, the change was not clinically significant. Additionally, levels of fatigue at 3, 6, 9, and 12-month post-SBRT were not significantly different from baseline. However, a racial disparity in fatigue following SBRT was present with African-American patients reporting increased treatment-related fatigue compared to Caucasian patients.

This is the first study to measure SBRT-related fatigue using the FACIT-Fatigue scale. The level of fatigue reported at baseline by prostate cancer patients [Mean (SD): 43.8 (8.2)] is very similar to that reported for the general US population [43.6 (9.4); Cella et al., 2002]. There was also no appreciable difference in baseline mean fatigue scores between Caucasian and African-Americans. Fatigue at 1-month post-SBRT in this patient population was very similar to what has been reported for non-anemic cancer patients [40.6 vs. 40.0; Cella et al., 2002].

Acute increases in fatigue following radiotherapy for prostate cancer have been previously reported (Stone et al., 2001; Hickok et al., 2005; Danjoux et al., 2007). The current results support the findings from previous studies that suggest fatigue peaks at 4–6 weeks after the initiation of radiation therapy. In most cases, these increases correspond to the completion of the extended radiotherapy regimen. However, hypofractionated SBRT treatment in the current study has a short, 1–2 week treatment duration. A limitation of this study is that fatigue levels were not measured at the end of SBRT. Thus, it is not known how fatigue levels at 1 week after SBRT initiation compare with those at 1-month post-SBRT. In addition, previous studies used scales other than FACIT-Fatigue to measure fatigue which limits direct comparison with our the results.

Long term fatigue was not observed in patients treated with SBRT. At 3 months follow-up fatigue among both Caucasian and African-American patients had returned to near baseline scores. Although there is some evidence that definitive radiotherapy is associated with long-term fatigue (at 12 months or more post-radiotherapy; Kyrdalen et al., 2010) studies looking at modern radiotherapy methods, such as intensity-modulated radiotherapy (IMRT) have not shown any lasting effects on fatigue or HRQOL measures (Marchand et al., 2010).

Previous studies have also reported that fatigue is associated with and has an impact on HRQOL (Rodrigues et al., 2004; Monga et al., 2005; Truong et al., 2006). For SBRT, no association or correlation of FACIT-Fatigue scores with the urinary, sexual, and bowel domains of EPIC-26 or with the AUA symptom score were observed. However, a strong positive correlation was observed for hormonal symptoms reported on EPIC-26 and the FACIT-Fatigue score. It is not clear if this is due to a hormonal basis for radiation-induced fatigue or a result of a similar item (“*lack of energy*” in EPIC and “*I have energy*” in FACIT-Fatigue) on both questionnaires. While small declines in testosterone levels have been reported following hypofractionated SBRT (Oermann et al., 2011), neither biochemical nor clinical hypogonadism appeared to result from the changes in testosterone level. Larger sample sizes are required to validate these observations.

Strengths of this study include a high proportion of African-Americans in the patient population, multiple follow-up intervals after SBRT, and use of validated instruments to measure fatigue and HRQOL. The FACIT-Fatigue Scale is a brief and easy to use measure of fatigue assessing the intensity of fatigue and the impact on a patient’s daily life. It distinguishes itself psychometrically from other measures as cutoff scores have been developed to aid in clinical interpretation. Additionally, raw scores have been transformed to a 0–100 interval measure which aids in the analysis of group differences assessing change over time as in the present study.

Limitations of this study include the number of participants, pre-existing co-morbidities and possible variation in patient lifestyle. Specifically, the statistical power was inadequate to analyze associations between fatigue and other baseline variables, such as PSA levels and tumor stage. In addition, almost all prostate cancer patients in our study had some co-morbidity, most frequently cardiovascular, and it is unknown whether changes in fatigue might have been associated with those co-morbidities. Nevertheless, the mean fatigue scores at baseline in our patient population were very similar to the general US population, thus it is unlikely that co-morbidities affected the reporting of fatigue levels. The multivariate analyses were adjusted for co-morbidities and none of the reported co-morbidities were associated with fatigue. Lastly, it is well known that lifestyle factors such as diet and exercise affect fatigue; however, lifestyle factors were not factored into the multivariate models.

A low incidence of fatigue was observed in prostate cancer patients undergoing hypofractionated SBRT. Reported fatigue was highest at 1 month post-SBRT, primarily among African-American patients. At 12 months post-SBRT fatigue levels for both Caucasians and African-American patients returned to near baseline levels. More studies with additional patients are needed to confirm these findings and investigate whether racial disparity is a component of fatigue following SBRT for prostate cancer.

## Conflict of Interest Statement

Sean Collins serves as a clinical consultant to Accuray, Inc. The other authors declare no competing interests.

## Footnotes

^1^www.facit.org.

^2^http://www.med.umich.edu/urology/research/EPIC/EPIC-26-Scoring-1.2007.pdf.
